# The Case against the Comforts Depot

**Published:** 1918-05-25

**Authors:** 


### The Case Against the Comforts Depot.

### THE ALLEGED ABUSE OF CHARITABLE FUNDS.

When voluntary agencies supplement State organisations it is not always easy to define their proper limit. The extras which they profess to supply sometimes include articles for which the State is responsible, and when the State actually supplies these there is excuse for criticism. On these lines a writer in the *Sheffield Telegraph* has criticised the local Soldiers' Comforts Depôt, which, he suggests, has supplied military hospitals with meat and bacon at a time when these supplies were or could have been drawn as army rations. The charge has a general interest, since all Comfort Funds are similarly tempted to supply things which should be drawn from official sources. It is true that rations on paper do not necessarily correspond with the rations on the plate. It is also true that those in charge of hospitals may be less active in drawing their full rations if they can get what they want from a local Comforts Fund. The criticisms directed against the Sheffield Soldiers' Comforts Depôt are detailed, and should be pondered elsewhere.

It is urged, for instance, that articles of the wounded soldier's kit lost through the action of the enemy, which are replaceable out of public funds, have been supplied by charity. Again, the supply of soldiers' shirts, which belongs to the Army Clothing Department, has apparently also been subsidised by voluntary funds. But the case of surgical dressings shows that it is not easy to draw a hard-and-fast line. They are an admitted necessity, and yet voluntary associations have helped to supply them from the first. We possess a Director-General of Voluntary Organisations. Is one of his duties to define the "extras" which Comforts Depôts may supply, and if so, is his policy to encourage them to subsidise the Government?

